# The expression of fibronectin is significantly suppressed in macrophages to exert a protective effect against *Staphylococcus aureus* infection

**DOI:** 10.1186/s12866-017-1003-9

**Published:** 2017-04-13

**Authors:** Hong-Yi Chen, Mei-Hui Lin, Chien-Cheng Chen, Jwu-Ching Shu

**Affiliations:** 1 Department of Medical Biotechnology and Laboratory Science, College of Medicine, Chang Gung University, No. 259, Wenhua 1st Road, Guishan, Taoyuan, 333 Taiwan; 2 Research Center for Pathogenic Bacteria, Chang Gung University, Taoyuan, Taiwan; 3 Department of Laboratory Medicine, Chang Gung Memorial Hospital, Taoyuan, Taiwan; 4 Department of Biotechnology, National Kaohsiung Normal University, Kaohsiung, Taiwan

## Abstract

**Background:**

Fibronectin (Fn) plays a major role in the attachment of *Staphylococcus aureus* to host cells by bridging staphylococcal fibronectin-binding proteins (FnBPs) and cell-surface integrins. A previous study demonstrated that the phagocytosis of *S. aureus* by macrophages is enhanced in the presence of exogenous Fn. We recently found that FnBPs overexpression also enhances phagocytic activity. The effect of *S. aureus* infection on the expression of macrophage Fn was investigated.

**Result:**

The level of Fn secreted by monocytes (THP-1), macrophages, human lung adenocarcinoma (A549) cells, and hepatocellular carcinoma (HepG2) cells in response to *S. aureus* infection was determined by Western blotting and it was significantly suppressed only in macrophages. The activation of signaling pathways associated with Fn regulation in macrophages and HepG2 cells was also investigated by Western blotting. Erk was activated in both macrophages and HepG2 cells, whereas Src-JNK-c-Jun signaling was only activated in macrophages. A significant decrease in macrophage viability was observed in response to *S. aureus* infection in the presence of exogenous Fn.

**Conclusion:**

The Src-JNK-c-Jun signaling pathway was activated in macrophages in response to *S. aureus* infection and resulted in the suppression of Fn expression. This suppression may play a protective role in macrophages against *S. aureus* infection. This study provides the first demonstration that Fn is suppressed in macrophages by *S. aureus* infection.

**Electronic supplementary material:**

The online version of this article (doi:10.1186/s12866-017-1003-9) contains supplementary material, which is available to authorized users.

## Background

*Staphylococcus aureus* is one of the leading causes of healthcare-associated and community-acquired infections [1]. This pathogen adheres to and invades host tissues mediated by the interaction between its surface adhesins and the host extracellular matrix (ECM). For example, bacterial attachment can be achieved through fibronectin (Fn) bridge between staphylococcal fibronectin-binding proteins (FnBPs) and the host’s cell-surface integrins [2]. We recently demonstrated that the expression of FnBP is upregulated by antibiotic stress, leading to an increase in cytotoxicity through an increase in bacterial attachment [3].

There are two principal types of Fn, the plasmatic and cell-surface-bound forms, which are generated from the same gene (*FN1*) transcript by alternative splicing [4]. Only the plasmatic form can bind to and be modified by FnBPs of which the process is essential for integrin binding [5]. This glycoprotein is an important component of the ECM and is functionally implicated in the regulation of several cellular processes, including cell adhesion, migration, as well as tissue repair and the recruitment of inflammatory cells [6]. Fn is majorly produced by hepatocytes, and part of its production is mediated by fibroblasts, monocytes, macrophages, and endothelial cells [7]. It has been reported that the expression of Fn is upregulated by NF-κB and downregulated by the c-Jun/c-Jun homodimer [8, 9]. The regulation of Fn expression is also associated with fibronectin-binding integrins through the modulation of Rho-GTP loading [10]. Several host cell signaling pathways such as focal adhesion kinase (FAK) and steroid receptor coactivator (Src), which are the intracellular tyrosine protein kinase, are activated by the formation of a Fn bridge that links FnBPs and integrins [11]. Once FAK is activated, downstream signaling pathways, such as those associated with MAPK-Erk kinases and Src-c-Jun NH2-terminal kinase (JNK)-c-Jun, which are key regulators controlling the expression of genes related to inflammation and cell adhesion, are also activated [12–14]. In addition, the expression level of plasma Fn is decreased and can be used as a biomarker for the evaluation of sepsis [15, 16]. It has been shown that the treatment of macrophages with lipopolysaccharide (LPS), the endotoxin of Gram-negative bacteria, leads to a decrease in Fn expression [16, 17]. However, the underlying mechanism remains unclear.

We recently found that the expression of FnBPs in *S. aureus* was increased after the application of the sub-lethal doses of cell wall active antibiotics [3]. This increased FnBP expression enhanced phagocytosis of *S. aureus* by macrophages, similarly to the effect obtained in other studies with the addition of exogenous Fn [18]. We proposed that the expression of Fn by macrophages would be increased in response to *S. aureus* infection to catch more bacterial cells, and this hypothesis was investigated in the present study. Unexpectedly, we found that the expression of Fn was significantly suppressed and the mechanism underlying was studied.

## Methods

### Bacterial strains

The allelic replacement of the *fnbAB* genes by a spectinomycin cassette (*spc*) was performed by introducing the pMAfnb plasmid into *S. aureus* strain ATCC12598 to generate the *fnbAB*-knockout strain SJC1221, according to a method described elsewhere [19]. The *spc* restricted by *Bam*HI and *Bgl*II was flanked by the upstream and downstream arms of the PCR product. The upstream arm originating from the 5′ end of *fnbA* was amplified by the primer pairs fnbA-F (5′-ATGACCATGGCTACTTGTCTTTGATCTCCGCTATTGT-3′) and fnbA-R (5′-AATTAGATCTCTTGTGTACAAGGGTTTCTGATGACTTG-3′) with *Bgl*II and *Nco*I restriction sites. The downstream arm was restricted by the primer pairs fnbB-F (5′-AGGATCCCTTCATAGTGTCATTG-3′) and fnbB-R (5′-GAGTCGACTGGTACAATCGAAG-3′) with *Bam*HI and *Sal*I restriction sites from the 3′ end of the *fnbB*. The constructed upstream arm-*spc*-downstream arm DNA fragment was then cloned into pMAD to yield pMAfnb.

### Cells

The human lung adenocarcinoma (A549) and hepatocellular carcinoma (HepG2) cell lines were maintained in DMEM, whereas the human acute monocytic leukemia (THP-1) cell line was maintained in RPMI 1640 medium. THP-1 cells (5 × 10^6^/ml) were differentiated using 10 ng/ml phorbol 12-myristate 13-acetate (PMA; Sigma-Aldrich, St. Louis, USA) for 2 days, and after the PMA-containing media was removed, the cells were incubated in fresh RPMI 1640 for an additional day [20]. All cell lines were cultured in serum-free media 1 day prior to bacterial infection. All the cell lines originally purchased from Bioresource Collection and Research Center (BCRC, Taiwan) were kindly given by Professor Hsin-Chih Lai in this department.

### Infection of cells

The bacterial infection of the cells was performed by inoculating with different *S. aureus* strains at a multiplicity of infection (MOI) of 25. Under specified conditions, Fn (200 or 400 μg/ml) was added to the medium to determine the macrophage viability after infection. Cells treated with LPS (100 μg/ml) were used as an experimental control. To determine the Fn-associated signaling in response to bacterial infection, the cells were pre-treated with several signaling inhibitors such as SP600125 (10 μM, JNK inhibitor), SB203580 (25 μM, p38 inhibitor), PP2 (25 μM, Src inhibitor), and PD98059 (25 μM, Erk kinase inhibitor) for 15 min prior to bacterial infection. Concentrations of those inhibitors used in this experiment were according to studies described elsewhere [21–24]. The infection was stopped at indicated time points, and the infected cells were washed twice with PBS. After the addition of gentamycin (50 μg/ml) and lysostaphin (10 μg/ml), the cells were incubated for an additional hour to kill and remove the extracellular bacteria. All the chemicals and antibiotics were purchased from Sigma-Aldrich.

### Western blotting

The level of secreted Fn and the activation of intracellularly Fn-associated signaling pathways were detected by Western blotting analysis using samples from cell culture supernatants and whole-cell lysates, respectively. Cells were pelleted by centrifugation, and supernatants containing the secreted Fn were removed and filtrated. The pelleted cells were lysed in lysis buffer as described elsewhere, and the concentration of protein in supernatant or cell lysate was determined [25]. Equal amounts of protein (3 μg/lane) were applied to SDS-PAGE gels. The membranes were probed with primary antibody (rabbit monoclonal) against fibronectin (Sigma-Aldrich), Src, Src-P, c-Jun, c-Jun-P (Ser73), p44/42 MAPK (Erk1/2), p44/42-P (Thr202/Tyr204) MAPK (Erk1/2), p38, p38-P (Thr180/Tyr182) or GAPDH (Cell Signaling Technology, Danvers, USA). After washing with TBST, the membranes were probed with secondary horse anti-rabbit HRP-conjugated antibody (Cell Signaling) for 1 h and developed using ECL reagents (Thermo Scientific, Waltham, USA). The blots were photographed and quantified by densitometry using ImageJ software. The results were presented as fold-change expression levels relative to that of the untreated cells and all of the samples were tested in three independent experiments.

### Reverse-transcription PCR

*S. aureus* infected or LPS treated macrophages were collected and pelleted 30 and 60 min post-infection and frozen on dry ice immediately. Total RNA was extracted from cell pellets using the TRIzol (Invitrogen, Waltham, USA) method followed by RQ1 RNase-free DNase (Promega, Madison, USA) treatment to eliminate any remaining DNA. mRNA levels of *FN1* were determined by RT-PCR with the iScript Reverse Transcription Supermix (Bio-Rad, Hercules, USA). The expression levels of *FN1* were normalized against the *GAPDH* expression level and quantified by densitometry using ImageJ software. The results were presented as fold-change expression levels relative to that of the untreated cells and all of the samples were tested in three independent experiments.

### Determination of cell viability

The viability of macrophages after *S. aureus* infection in the presence of exogenous Fn was determined using the MTT assay with MTT (3- (4,5-cimethylthiazol-2-yl)-2,5-diphenyl tetrazolium bromide; 0.5 mg/ml; Sigma-Aldrich), as described previously [26]. Four hours post-infection, 100 μl of MTT solution was added to each well, and the plates were incubated at 37 °C for 4 h. The purple formazan crystals were dissolved by adding 100 μl of DMSO, and the absorbance at A_570_ was spectrophotometrically measured with a reference wavelength of A_690_. The results are expressed as the percent absorbance of each experimental well as a function of the absorbance of the well containing untreated cells. Three wells were measured for each experimental condition, and each experimental condition was tested in three independent experiments. The attached viable cells were observed by crystal violet staining following to 1 h gentamycin and lysostaphin treatment to eliminate the extracellular bacteria. The cells were fixed by 1% formaldehyde followed by staining with PBS containing 0.5% crystal violet solution (Sigma-Aldrich) for 20 min at room temperature. The cells were gently washed with deionized water and air-dried. The attached damage cells were also visualized by propidium iodide (PI) staining with a commercial apoptosis detection kit (BioVision, Milpitas, USA) by microscopy according to the manufacturer’s instructions.

### Statistical analysis

A one-way ANOVA with Games-Howell post-hoc test was used to analyze the experimental data and to compare means. Error bars in the bar graphs of the relative densitometric quantification or cell viability represent as the standard deviation from three experimental repetitions. *P*-values of less than 0.05 were considered statistically significant.

## Results

### Expression of fn by macrophages was suppressed by *S. aureus* infection

**Fig. 1:**
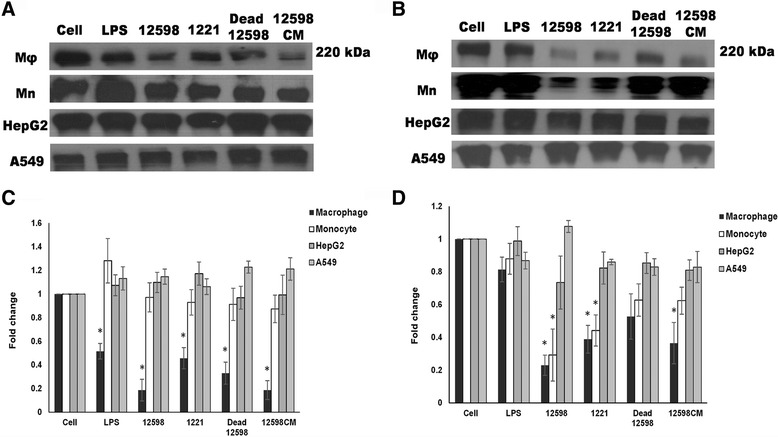
Expression of secreted Fn is significantly suppressed in macrophages after *S. aureus* infection. The levels of Fn secreted by THP-1-derived microphages (Mφ), monocytes (Mn), hepatocytes (HepG2), or alveolar basal epithelial cells (A549) after (**a**) 2 h and (**b**) 24 h of *S. aureus* infection were determined and the representative Western blot images are shown. The relative densitometric quantification of above results is shown in (**c**) and (**d**), respectively. The numbers ‘12598’ and ‘1221’ represent the *S. aureus* strains ATCC 12598 and SJC1221 (FnBP deficient), respectively. Other abbreviations: 12,598 dead, heat-killed strain 12,598; 12,598 CM, filtered culture medium containing bacterial exotoxins. LPS was used as an experimental control. The fold change of each sample was compared with untreated cells for each cell type. * *P* < 0.05

Fn plays an important role in bridging the staphylococcal FnBPs to the cell-surface α_5_β_1_ integrins. The effect of *S. aureus* infection on the expression of Fn by macrophages was investigated. A decrease in secreted Fn expression was observed in macrophages after both 2 and 24 h (5.3- and 4.0-fold decreased, respectively) of *S. aureus* 12,598 infection (Fig. 1). Equal protein loading across all gel lanes was visualized via the Coomassie blue staining (Additional file 1: Figure S1). To study the role of staphylococcal FnBP on macrophage Fn expression, macrophages were infected with the FnBP-deficient strain SJC1221. A mild decrease in Fn expression (2.2- and 2.6-fold decreased 2 and 24 h post-infection, respectively and the same hereafter) was observed suggesting that the FnBP-mediated bacteria-host interaction contributes, at least partly, to the reduction in Fn. There was no difference in the bacterial growth between 12,598 and SJC1221, indicating that the mitigation of the reduction of Fn expression observed with the mutant strain is not due to the bacterial load (Additional file 2: Figure S2). The effect of bacterial cell wall components on the reduction of Fn secretion was investigated through the incubation of heat-killed *S. aureus* with macrophages, and a reduction in Fn secretion (3.0- and 1.9-fold) was also observed. The filtered supernatant from an overnight bacterial culture was then incubated with macrophages to study the effect of bacterial pathogen-associated molecular patterns (PAMPs) such as lipopeptides, bacterial DNA, and exotoxins, particularly hemolysins, on the suppression of Fn secretion. A suppression of Fn secretion was also detected (5.6- and 2.8-fold), suggesting that the Fn secretion suppressed by *S. aureus* is multifactorial (Fig. 1). To investigate whether the *S. aureus*-mediated reduction in Fn secretion was unique to macrophages, the level of Fn secreted by different types of cells, including monocytes (macrophage precursors), A549 cells, and HepG2 cells, after *S. aureus* infection was determined. The level of Fn by these cells was not significantly changed as was observed with macrophages throughout the period of this experiment except for monocytes 24 h post-infection, probably due to the monocyte-to-macrophage differentiation upon bacterial infection (Fig. 1). Morphological characteristics (pseudopodia) of macrophages were also observed under light field (Additional file 3: Figure S3).

**Fig. 2:**
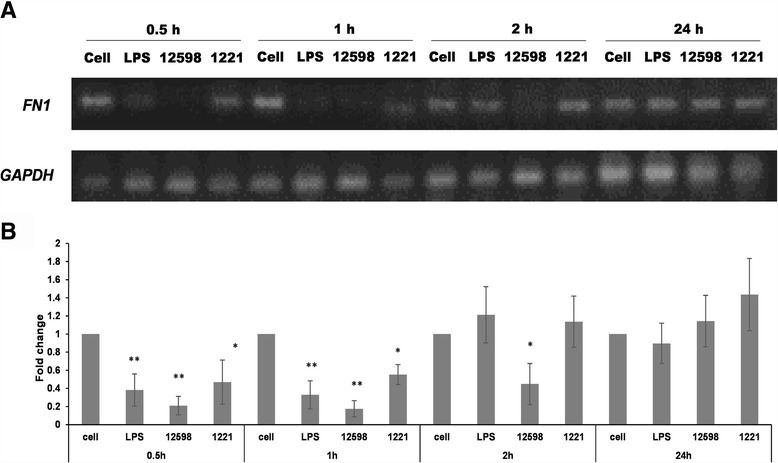
Expression of *FN1* is suppressed in macrophages after *S. aureus* infection. **a** The level of *FN1* expression in macrophages after *S. aureus* infection was determined time-coursely by RT-PCR and the representative images are shown. **b** The relative densitometric quantification of above results is shown. The numbers ‘12598’ and ‘1221’ represent the *S. aureus* strains ATCC 12598 and SJC1221 (FnBP deficient), respectively. The fold change of each transcript was compared with untreated cells at each time point and was indicated. * *P* < 0.05; ** *P* < 0.01

Transcriptional level of *FN1* in macrophages upon infection was also determined by RT-PCR. Reduction of the expression of *FN1* was observed when macrophages were infected by strain 12,598 and the strongest reduction was observed at 30 min post-infection. Such reduction was mitigated at 2 h post-infection when macrophages were incubated with strain SJC1221 or LPS (Fig. 2).

### Reduction in fn expression in macrophages was mediated by c-Jun phosphorylation through the activation of Src signaling

**Fig. 3:**
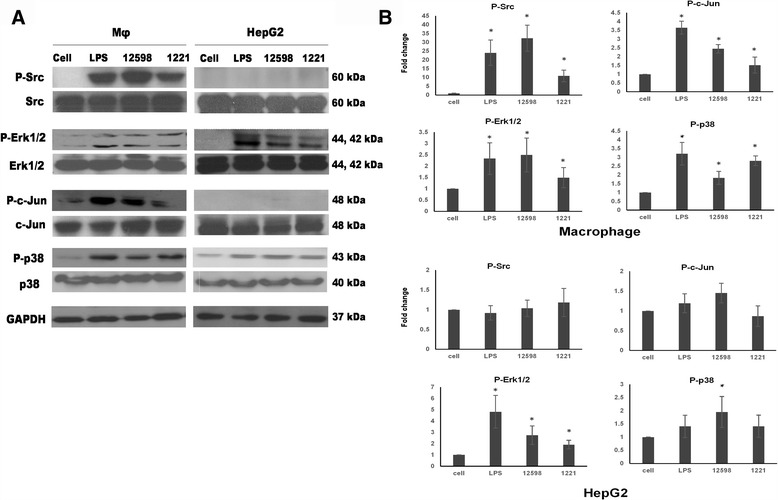
Activation of c-Jun through Src signaling is triggered in macrophages upon *S. aureus* infection. **a** The activation of the Src-JNK-c-Jun, MEK-Erk, and p38 signaling pathways was investigated by determining the expression levels of phosphorylated Src, c-Jun, p38, and Erk, respectively, by Western blotting. Samples were collected 1 h post-infection. **b** The relative densitometric quantification of above results is shown to indicate the fold change of protein levels relative to untreated cells after normalization to GAPDH. * *P* < 0.05

As described above, the expression of Fn in cancer cells is downregulated by c-Jun homodimers, the activation of which is regulated through Src-JNK signaling. Fn is upregulated by c-Jun/c-Fos, the AP-1 heterodimer, upon the activation of p38 MAPK [13]. The activation of Src has been shown to be associated with integrins, and a weaker suppression of Fn was found in macrophage infected with the *S. aureus* FnBP mutant strain. These results suggest that Src signaling may be crucial to the suppression of Fn expression in *S. aureus*-infected macrophages. In addition, c-Jun has also been shown to be regulated by Erk [27, 28]. Therefore, the activation of Src, Erk, p38, and c-Jun in macrophages and HepG2 cells after *S. aureus* infection was then investigated and compared. The results shown in Fig. 3 indicate that Src, Erk, and p38 were activated (phosphorylated) in macrophages after infection with *S. aureus* 12,598. Activation of c-Jun was also detected, indicating the subsequent activation of JNK. A similar result was observed in macrophages treated with LPS. A weaker activation of Src and c-Jun but not Erk was detected when the FnBP-deficient strain SJC1221 was employed (Fig. 3). However, with the exception of Erk and p38, Src and c-Jun were not activated in HepG2 cells, in contrast to the results observed in macrophages subjected to the same treatment (Fig. 3). The finding that both Erk and p38 were activated in both cell lines suggests that the activation of Erk and p38 induced by *S. aureus* infection may not play a role in the c-Jun-mediated suppression of Fn. We further investigated the effect of PAMPs and exotoxins secreted to the medium on the fibronectin-associated signaling activation between macrophages and HepG2. A similar result, except for weak activation of Erk in untreated HepG2, was observed as that found when cells were infected with filtered supernatant from an overnight bacterial culture (Additional file 4: Figure S4).

**Fig. 4:**
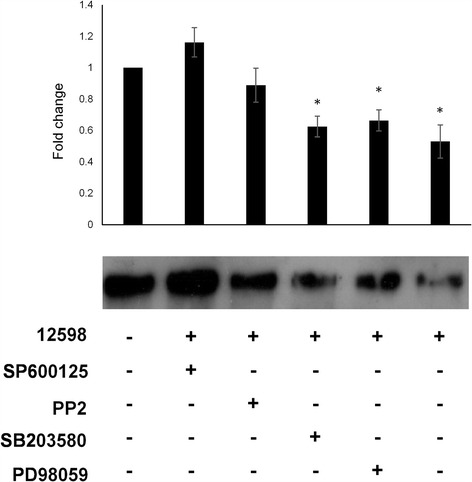
Suppression of Fn is abrogated to the basal level by pre-treatment with a Src or JNK inhibitor. Macrophages were pre-treated with JNK (SP600125), Src (PP2), p38 (SB203580), or Erk (PD98059) inhibitors prior to infection with strain 12,598. The level of secreted Fn 2 h post-infection was determined by Western blotting and the relative densitometric quantification is shown on the top. The fold change of each sample was compared with untreated cells. * *P* < 0.05

To further confirm whether Src-JNK-c-Jun signaling plays a major role in suppressing Fn expression after *S. aureus* infection, four signaling inhibitors were applied prior to infection. The treatment with inhibitors alone had no significant effect on Fn expression among cell controls, but Src and p38 inhibitors showed cytotoxicity to macrophages (Additional file 5: Figure S5 and Additional file 6: Figure S6). The suppression of Fn in macrophages by *S. aureus* infection was abrogated to the basal level by pre-treatment either with the Src (PP2) or JNK inhibitor (SP600125), whereas Fn suppression was not affected by pre-treatment with the Erk (PD98059) or p38 (SB203580) inhibitors (Fig. 4). This result suggests that the Src-JNK-c-Jun signaling pathway plays a major role in the suppression of Fn.

### Viability of macrophages after *S. aureus* infection was decreased in the presence of exogenous fn

**Fig. 5:**
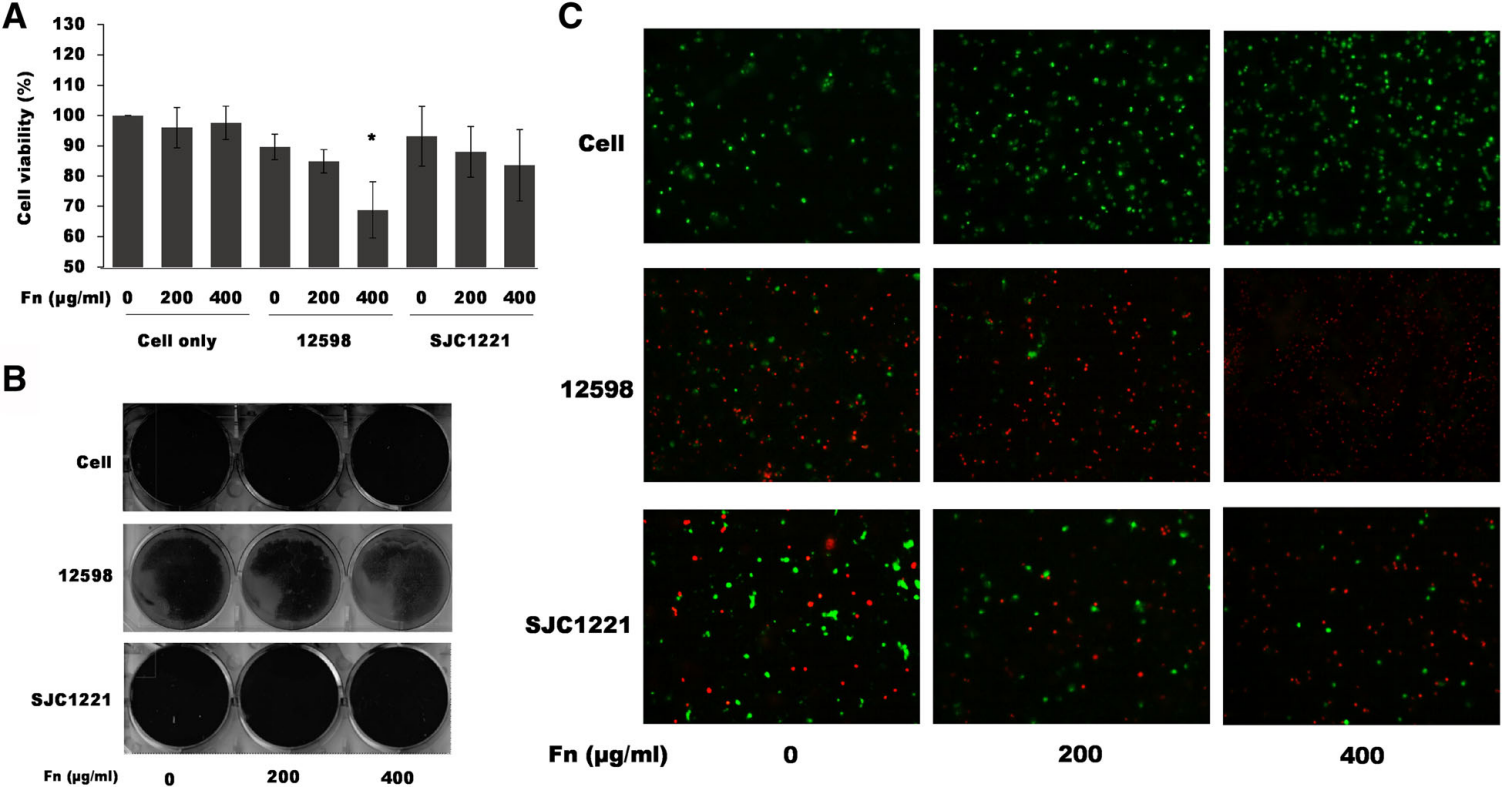
Macrophage viability was decreased after *S. aureus* infection in the presence of exogenous Fn. The viability of macrophages after infection (4 h) with strain 12,598 or SJC1221 in the presence of exogenous Fn was determined by (**a**) the MTT assay, or visualized either by (**b**) crystal violet staining or (**c**) PI staining. Damaged cells were observed by detecting fluorescent PI (red) under a fluorescence microscope with the magnification of 100×. Cell viability was defined as 100% when cells were cultured in medium only (untreated). * *P* < 0.05

The physiological role of Fn suppression in macrophages after *S. aureus* infection was investigated by the addition of exogenous Fn followed by the evaluation of cell viability using the MTT assay and crystal violet staining. The concentration of plasma Fn is 300 to 400 μg/ml [16]. Therefore, the concentrations of Fn used in our experiment were 200 and 400 μg/ml. In comparison with the Fn-untreated macrophages, a significant decrease (approximately 30%) in cell viability was observed after infection with strain 12,598 in the presence of 400 μg/ml Fn. No obvious difference in cell viability was detected when the macrophages were challenged with the FnBP-deficient strain SJC1221 in the presence of either 200 or 400 μg/ml Fn (Fig. 5a). A similar result was observed when the viable macrophages were visualized by crystal violet staining (Fig. 5b). It is also highly likely that the crystal violet will stain both the mammalian and bacterial cells. Therefore, a PI staining was employed to indicate the cell viability (damage). Results shown in Fig. 5c indicate that more and more strain 12,598-infected macrophages were damaged (red color) with the increase in the concentration of exogenous Fn and such damage was mitigated when strain SJC1221 was used. This result suggests that the reduction in Fn expression in macrophages after *S. aureus* infection may play a protective role against *S. aureus* infection.

## Discussion

In this present study, the expression level of secreted Fn was found to be significantly suppressed in macrophages by *S. aureus* infection. This suppression has also been observed in macrophages treated with either exotoxins or heat-killed *S. aureus*, indicating that the Fn suppression mediated by *S. aureus* is multifactorial. A mitigation of Fn suppression was detected in the co-cultured of FnBP-deficient *S. aureus* strain with macrophages, implying that the FnBP-mediated bacteria-host interaction may contribute to the Fn suppression observed in macrophages. Fn suppression in macrophages was found to be mediated only through the activation of the Src-JNK-c-Jun signaling pathway. The physiological role underlying Fn suppression may reduce the cytotoxicity caused by *S. aureus* infection. To the best of our understanding, this study provides the first demonstration of Fn suppression in macrophages upon bacterial infection, at least by *S. aureus*.

**Fig. 6:**
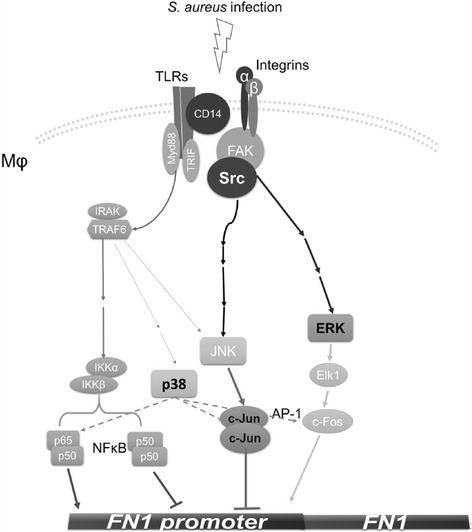
Schematic diagram of the regulation of Fn expression in macrophages after *S. aureus* infection. The expression of Fn is upregulated by the NF-κB p50/p65 heterodimer through the activation of the TLR-MyD88 signaling pathway. Fn suppression is achieved by the expression and activation of the c-Jun homodimer through the activation of the FAK-Src-JNK signaling pathway after *S. aureus* infection. The association of FAK with TLRs/CD14 is required for the activation of Src

Fn plays an important role in bridging the connection between *S. aureus* FnBP and cell integrins. Integrins are cell-surface glycoprotein composed of α and β subunits that are involved in the formation of the cell ECM and in the mediation of ECM and pathogen interactions [7, 10, 29]. These proteins have been identified to serve as receptors that trigger several signaling pathways in response to bacterial infection [5, 12, 30, 31]. The pathways investigated in this study are summarized in Fig. 6. The expression of c-Jun or c-Fos has been demonstrated to be associated with the suppression of Fn in cancer cells [9, 32]. The activation of the FAK-associated protein complex plays a central role in controlling the downstream signaling pathways, including c-Jun and c-Fos regulation [29]. Upon stimulation, such as bacterial infection, the Erk-Elk1-c-Fos, Src-JNK-c-Jun, and p38 signaling pathways are activated. However, an association with Toll-like receptors (TLRs) and CD14 is required for the activation of Src in addition to the FAK complex [31, 33]. In contrast, TLR-MyD88-NF-κB signaling is involved in the upregulation of Fn when p65/p50 heterodimers are bound to the *FN1* promoter [34]. The formation of p50/p50 homodimers plays a minor role in downregulating Fn expression [35].

*S. aureus* infection-mediated Fn suppression was only observed in macrophages among the distinct types of cells employed in this study (Fig. 1). The activation of Erk, p38 and Src-JNK-c-Jun signaling pathways in macrophages and hepatocytes (HepG2) was then investigated. The activation of Erk and p38 was detected in both cells, whereas Src-JNK-c-Jun signaling was only activated in macrophages upon *S. aureus* infection (Fig. 3). The expression of Fn was not altered in the presence of Erk and p38 inhibitors, suggesting that both signaling are not involved in the suppression of Fn. However, the expression of Fn was recovered to a basal level by pre-treatment with a JNK inhibitor, indicating that the observed Fn suppression is mediated through the Src-JNK signaling (Fig. 4). Though Src inhibitor (PP2) showed cytotoxicity against macrophages but expression level of Fn was still higher than PP2-free counterpart indicating that the abrogation of the Fn suppression by PP2 was not due to the cell death. It has been demonstrated that α-hemolysin triggered the activation of FAK and Src signaling through the mediation of integrins [36]. Therefore, the Fn suppression as also observed when macrophages were incubated with bacterial culture medium (Fig. 1 and Additional file 4: Figure S4). *S. aureus* infection plays distinct roles in Fn expression in macrophages and others cells, and this difference is likely due to differences in CD14 expression. CD14 is expressed mainly by macrophages and dendritic cells and acts as a co-receptor together with TLRs for the recognition of PAMPs, such as LPS [33, 37, 38]. LPS, which was used as an experimental control in our study, has been shown to downregulate Fn expression in macrophages, but the underlying mechanism remains unclear and no follow-up study has been reported [17]. LPS also triggers Src signaling and the accumulation of c-Jun, leading to the overexpression of cytokines [39, 40]. As described above, the association of TLRs, CD14, and the FAK complex is required for Src activation [31, 33]. Nevertheless, LPS was used as an experimental technique control and the difference of the suppressive mechanism between *S. aureus* infection and LPS treatment should be further elucidated.

Through the time course study, the strongest suppression of *FN1* transcription and secreted Fn expression was observed at 30 min and 2 h-post infection, respectively (Figs. 1 and 2). We may not rule out that the decreased Fn expression was partially due to the effects on cell viability. Only approximately 10% of the macrophages had been killed following to *S. aureus* infection (4 h post-infection; Fig. 5a) but a five-fold reduction of the Fn secretion was already observed 2 h post-infection (Fig. 1), as well as the downregulation of the *FN1* transcription was observed (Fig. 2). Suppressed Fn expression upon *S. aureus* infection was only observed in macrophages, not in other cell types, indicating that cell viability had less effect on the Fn expression (Fig. 1). In addition, equal amount of the total exoproteins were loaded per sample for Western blotting (Additional file 1: Figure S1) and the relatively low levels of Fn expression was not caused from the decreased sample loading.

The interesting finding in this study is that the suppression of FN expression in macrophages may play a protective role against *S. aureus* infection (Fig. 5). The suppression of Fn may reduce the attachment of bacteria to macrophages through the FnBP-Fn-integrin connection. Therefore, the addition of exogenous Fn had no significant effect on the macrophage viability upon infection with FnBP deficient strain SJC1221. Previous studies have demonstrated that the production of TNF-α was increased from macrophages infected by group B streptococci in the presence of exogenous Fn [41]. A large release of TNF-α has been shown to be associated with harmful pathophysiological effects and increased mortality from septic shock [42]. In addition, the abnormally diminished concentration of plasma Fn has been used as a biomarker in evaluating sepsis [15]. The reduction in the plasma Fn concentration observed in patients with sepsis may arise from intravascular coagulation and cryofibrinogenemia, which is likely due to increased plasma Fn catabolism secondary to the intravascular formation of fibrin [15]. However, the mechanisms underlying this reduction remain unclear. Therefore, a reduction in the Fn concentration may be beneficial to the host, at least to macrophages, after *S. aureus* infection. Macrophages are majorly found in tissues through the differentiation of monocytes when they leave the blood. Thus, such protective mechanisms may be relevant to localized infection in the tissues. However, Fn is also present in the tissues and the effect of tissue Fn on the macrophages against *S. aureus* infection remains to be elucidated further. An animal model which reflects tissue infection for evaluating the local Fn expression level by immunofluorescent assay can be conducted. In addition, fibroblasts are widely dispersed in connective tissues and the effect of *S. aureus* infection on the expression of Fn by fibroblasts can be studied in the future.

## Conclusions

Our study provides the first demonstration that Fn is suppressed in macrophages by *S. aureus* infection. This *S. aureus* infection induced suppression of Fn expression from macrophages was mediated by Src-JNK-c-Jun signaling pathway. This suppression may play a protective role in macrophages against *S. aureus* infection.

## Additional files

Additional file 1: Figure S1. Loading controls of exoproteins. Equal protein loading across all gel lanes was visualized via the Coomassie blue staining for experimental results shown in (A) Fig. 1, (B) Fig. 4, and (C) Additional file 5: Figure S5. (TIFF 3956 kb)

Additional file 2: Figure S2. Comparison of the viable colony counts between *S. aureus* strain ATCC 12598 and its *fnbAB*-knockout mutant SJC1221. Similar colony counts were found for these two strains throughout a 24-h culture. (TIFF 33 kb)

Additional file 3 Figure S3. Morphological characteristic of macrophages differentiated from monocytes was observed after *S. aureus* infection. Morphology of monocytes was visualized under light field (400×) after (A) 2 h and (B) 24 h of filtered supernatant from an overnight *S. aureus* 12,598 bacterial culture. Cells with pseudopodia, morphological characteristic of macrophages, were observed (arrow) 24 h post-infection. (TIFF 2850 kb)

Additional file 4: Figure S4. Activation of c-Jun through Src signaling is triggered in macrophages upon infection with secreted PAMPs and exotoxins. (A) Macrophages or HepG2 cells were incubated with strain 12,598 overnight culture medium for 2 h. The activation of the Src-JNK-c-Jun, MEK-Erk, and p38 signaling pathways was investigated by determining the expression levels of phosphorylated Src, c-Jun, p38, and Erk, respectively, by Western blotting. (B) The relative densitometric quantification of above results is shown to indicate the fold change of protein levels relative to untreated cells after normalization to GAPDH. * *P* < 0.05. (TIFF 842 kb)

Additional file 5: Figure S5. The effect of different signaling inhibitors on the Fn expression of macrophages. Macrophages were treated with JNK (SP600125), Src (PP2), p38 (SB203580), or Erk (PD98059) inhibitors prior to the determination of the level of secreted Fn by Western blotting and the relative densitometric quantification is shown on the top. The fold change of each sample was compared with untreated cells. (TIFF 282 kb)

Additional file 6 Figure S6. Cytotoxicity of different signaling inhibitors to the (A) macrophages and (B) *S. aureus* strains. Survival of macrophages or *S. aureus* strains ATCC12598 and SJC1221 was evaluated using MTT assay upon treatment with different signaling inhibitors for 24 h. A one-way ANOVA with Games-Howell post-hoc test was used to analyze. Error bars in the bar graphs of the relative densitometric quantification or cell viability represent as the standard deviation from three experimental repetitions. Cell viability was defined as 100% when cells were cultured in serum-free medium. * *P* < 0.05. (TIFF 94 kb)

## Abbreviations

A549: Human lung adenocarcinoma cells
ECM: Extracellular matrix
FAK: Focal adhesion kinase
Fn: Fibronectin
FnBPs: Fibronectin-binding proteins
HepG2: Hepatocellular carcinoma cells
LPS: Lipopolysaccharide
MOI: Multiplicity of infection
*Spc*: Spectinomycin cassette
Src: steroid receptor coactivator
THP-1: human acute monocytic leukemia
TLRs: Toll-like receptors
