# Supplementary figures and images for: The expression of fibronectin is significantly suppressed in macrophages to exert a protective effect against Staphylococcus aureus infection
Source: BMC Microbiol. 2017 Apr 13;17:92. doi: 10.1186/s12866-017-1003-9 (PMC5390343; doi:10.1186/s12866-017-1003-9)

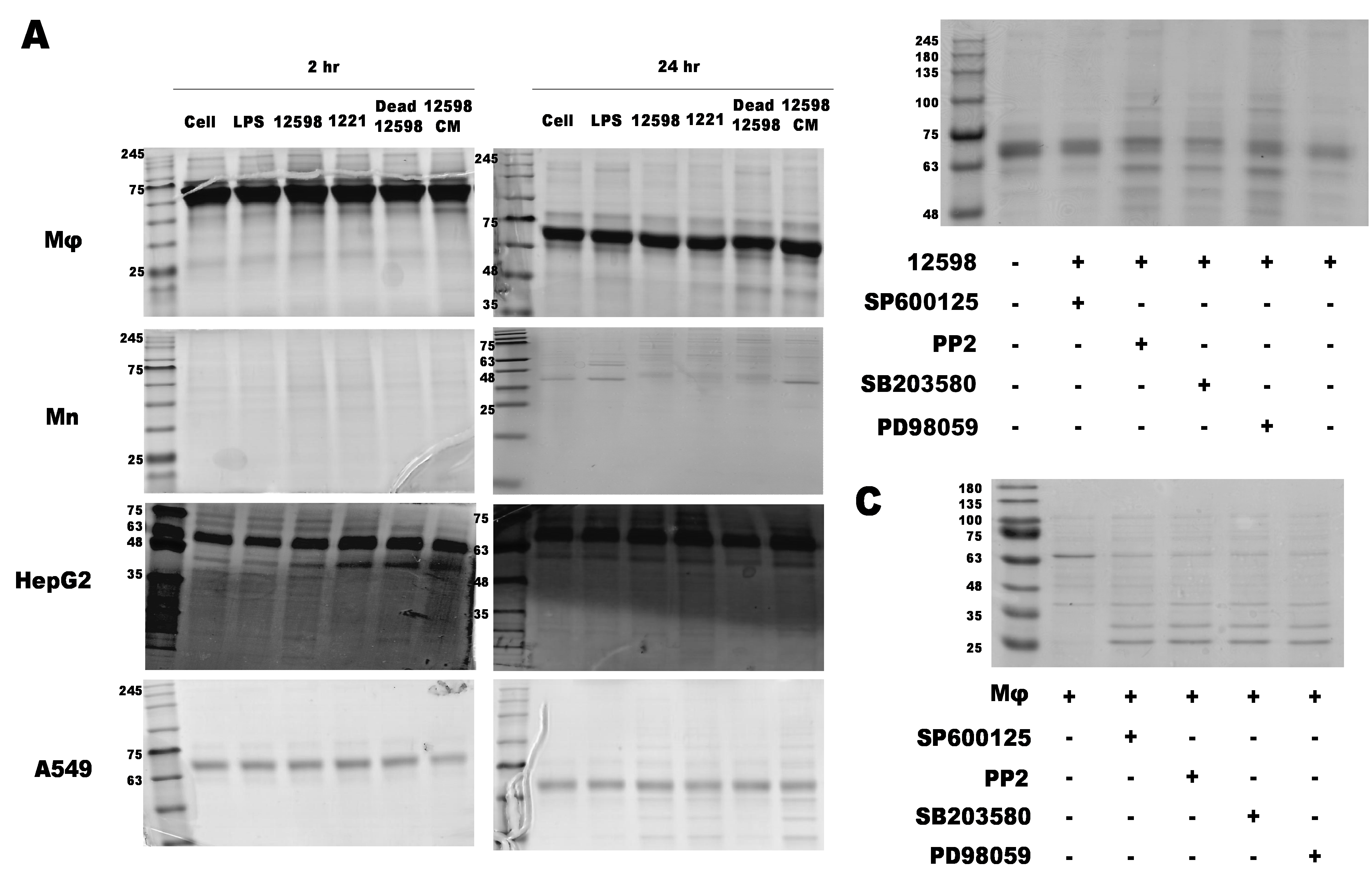

Supplement: Supplementary file 1 — Loading controls of exoproteins. Equal protein loading across all gel lanes was visualized via the Coomassie blue staining for experimental results shown in (A) Fig. 1, (B) Fig. 4, and (C) Additional file 5: Figure S5. (TIFF 3956 kb) [file 12866_2017_1003_MOESM1_ESM.tif]

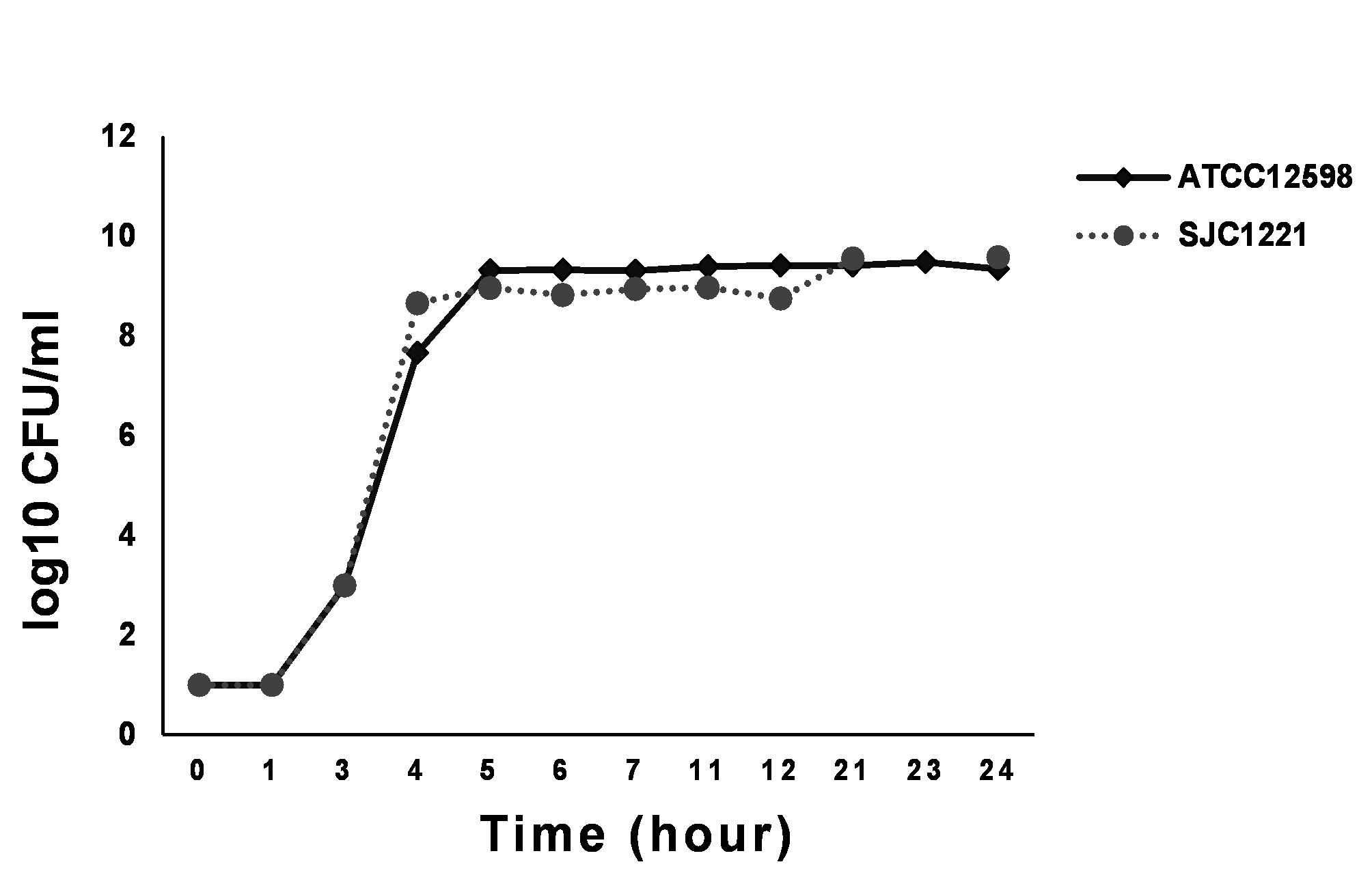

Supplement: Supplementary file 2 — Comparison of the viable colony counts between S. aureus strain ATCC 12598 and its fnbAB-knockout mutant SJC1221. Similar colony counts were found for these two strains throughout a 24-h culture. (TIFF 33 kb) [file 12866_2017_1003_MOESM2_ESM.tif]

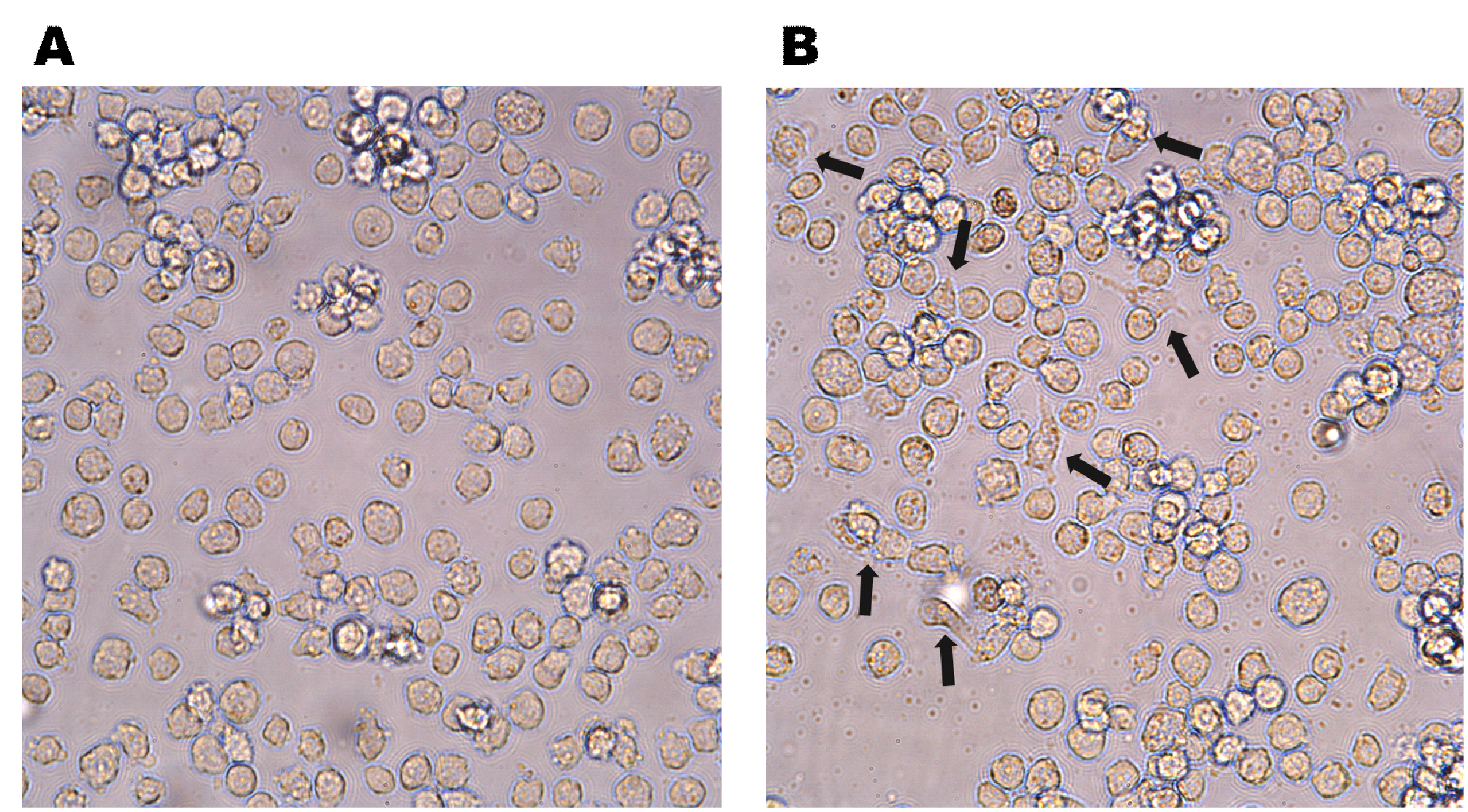

Supplement: Supplementary file 3 — Morphological characteristic of macrophages differentiated from monocytes was observed after S. aureus infection. Morphology of monocytes was visualized under light field (400×) after (A) 2 h and (B) 24 h of filtered supernatant from an overnight S. aureus 12,598 bacterial culture. Cells with pseudopodia, morphological characteristic of macrophages, were observed (arrow) 24 h post-infection. (TIFF 2850 kb) [file 12866_2017_1003_MOESM3_ESM.tif]

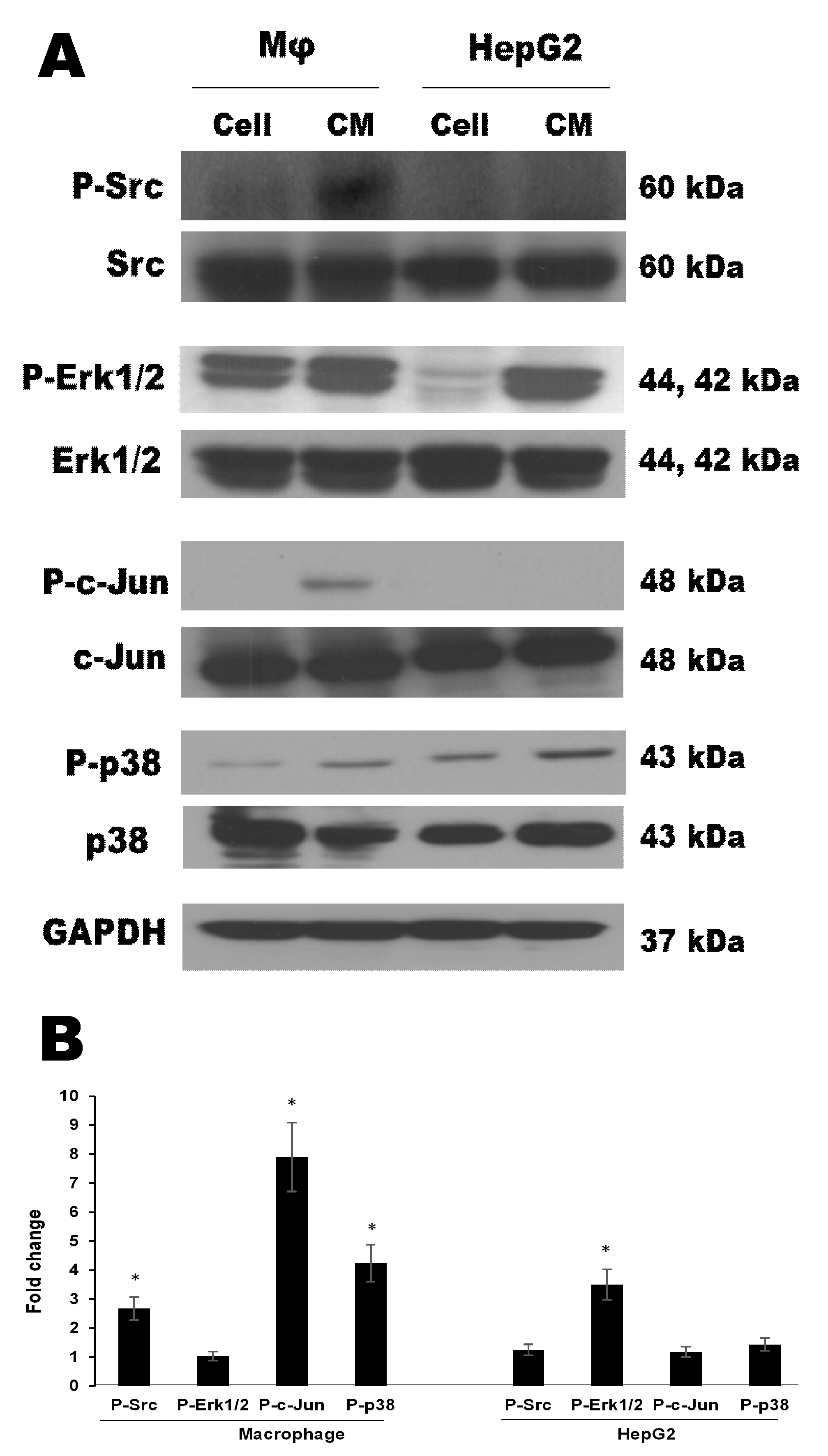

Supplement: Supplementary file 4 — Activation of c-Jun through Src signaling is triggered in macrophages upon infection with secreted PAMPs and exotoxins. (A) Macrophages or HepG2 cells were incubated with strain 12,598 overnight culture medium for 2 h. The activation of the Src-JNK-c-Jun, MEK-Erk, and p38 signaling pathways was investigated by determining the expression levels of phosphorylated Src, c-Jun, p38, and Erk, respectively, by Western blotting. (B) The relative densitometric quantification of above results is shown to indicate the fold change of protein levels relative to untreated cells after normalization to GAPDH. * P < 0.05. (TIFF 842 kb) [file 12866_2017_1003_MOESM4_ESM.tif]

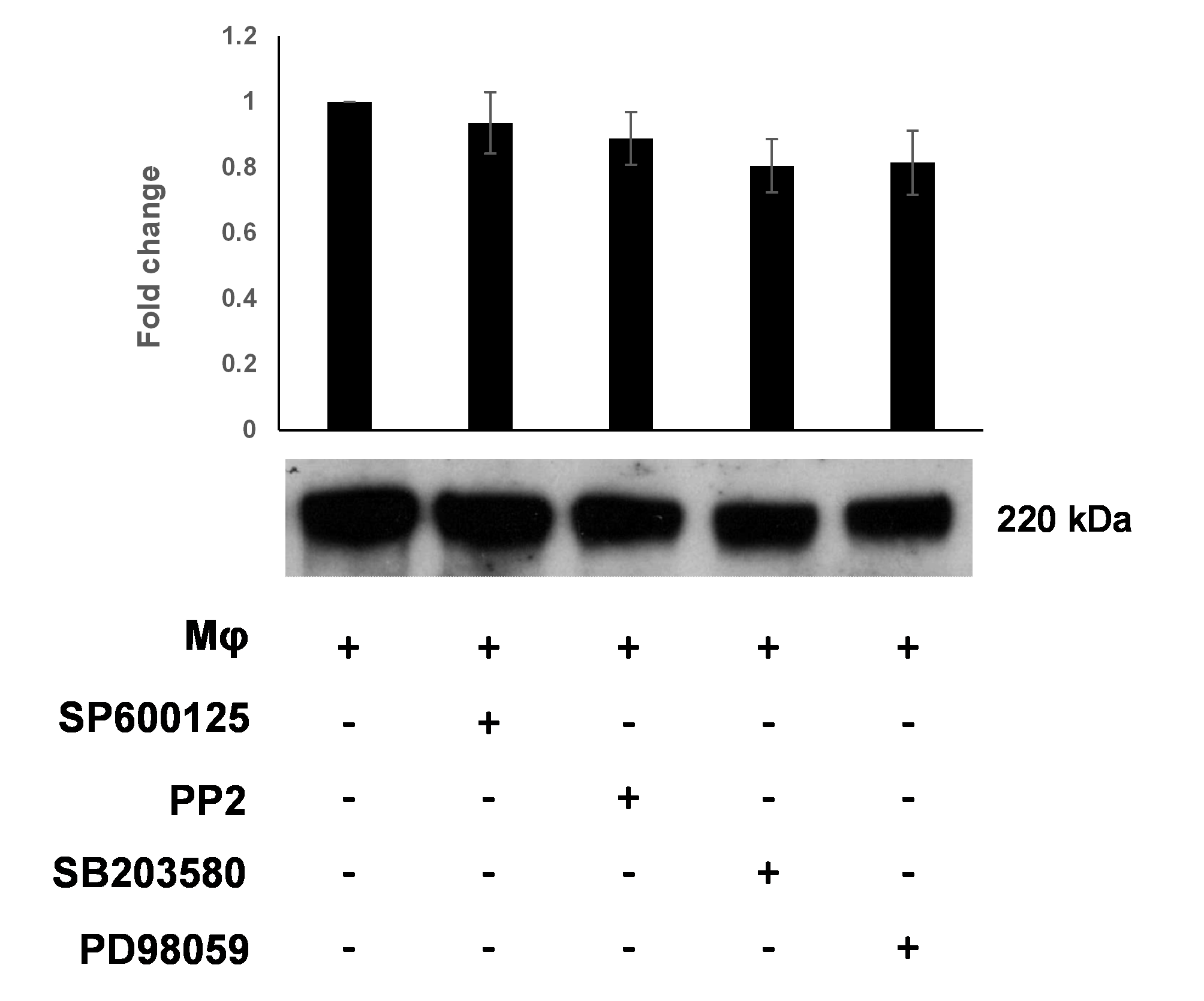

Supplement: Supplementary file 5 — The effect of different signaling inhibitors on the Fn expression of macrophages. Macrophages were treated with JNK (SP600125), Src (PP2), p38 (SB203580), or Erk (PD98059) inhibitors prior to the determination of the level of secreted Fn by Western blotting and the relative densitometric quantification is shown on the top. The fold change of each sample was compared with untreated cells. (TIFF 282 kb) [file 12866_2017_1003_MOESM5_ESM.tif]

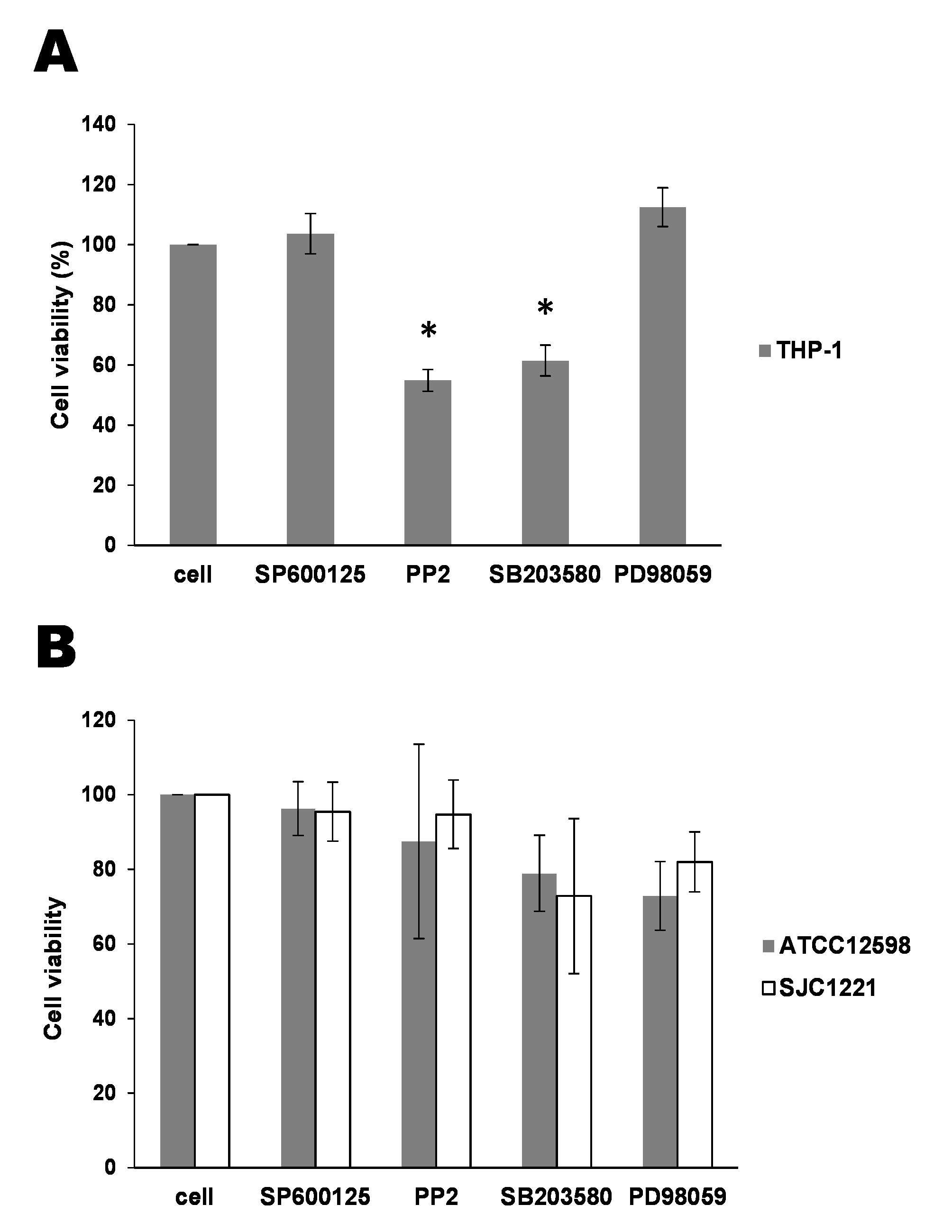

Supplement: Supplementary file 6 — Cytotoxicity of different signaling inhibitors to the (A) macrophages and (B) S. aureus strains. Survival of macrophages or S. aureus strains ATCC12598 and SJC1221 was evaluated using MTT assay upon treatment with different signaling inhibitors for 24 h. A one-way ANOVA with Games-Howell post-hoc test was used to analyze. Error bars in the bar graphs of the relative densitometric quantification or cell viability represent as the standard deviation from three experimental repetitions. Cell viability was defined as 100% when cells were cultured in serum-free medium. * P < 0.05. (TIFF 94 kb) [file 12866_2017_1003_MOESM6_ESM.tif]
